# A theoretical account of cue averaging in the rodent head direction system

**DOI:** 10.1098/rstb.2013.0283

**Published:** 2014-02-05

**Authors:** Hector J. I. Page, Daniel M. Walters, Rebecca Knight, Caitlin E. Piette, Kathryn J. Jeffery, Simon M. Stringer

**Affiliations:** 1 Oxford Centre for Theoretical Neuroscience and Artificial Intelligence, Departmental of Experimental Psychology, University of Oxford, South Parks Road, Oxford OX1 3UD, UK; 2 Division of Psychology and Language Sciences, Department of Cognitive, Perceptual and Brain Sciences, Institute of Behavioural Neuroscience, University College London, 26 Bedford Way, London WC1H 0AP, UK

**Keywords:** path integration, head direction cells, isomapping, sensory cue integration, attractor dynamics, neural networks

## Abstract

Head direction (HD) cell responses are thought to be derived from a combination of internal (or idiothetic) and external (or allothetic) sources of information. Recent work from the Jeffery laboratory shows that the relative influence of visual versus vestibular inputs upon the HD cell response depends on the disparity between these sources. In this paper, we present simulation results from a model designed to explain these observations. The model accurately replicates the Knight *et al.* data. We suggest that cue conflict resolution is critically dependent on plastic remapping of visual information onto the HD cell layer. This remap results in a shift in preferred directions of a subset of HD cells, which is then inherited by the rest of the cells during path integration. Thus, we demonstrate how, over a period of several minutes, a visual landmark may gain cue control. Furthermore, simulation results show that weaker visual landmarks fail to gain cue control as readily. We therefore suggest a second longer term plasticity in visual projections onto HD cell areas, through which landmarks with an inconsistent relationship to idiothetic information are made less salient, significantly hindering their ability to gain cue control. Our results provide a mechanism for reliability-weighted cue averaging that may pertain to other neural systems in addition to the HD system.

## 1. Introduction

Single-cell recordings in the limbic system of rats and primates reveal the presence of head direction (HD) cells, which respond to the animal's HD in the horizontal (azimuth or yaw) plane [1,2]. Individual HD cells have a Gaussian firing profile of approximately 90° width, centred on a preferred HD to which the cell maximally responds. Outside this Gaussian tuning curve, the cell firing is at or near zero [3,4].

HD cell firing is influenced by both descending visual input and ascending vestibular input. Vestibular information appears to be essential for the HD signal, as damage to, or inactivation of, vestibular end organs eliminates directional firing in the anterior dorsal thalamic nucleus and postsubiculum [5]. HD cells continue to fire in the absence of visual input [6] and update this firing using vestibular input to reflect the true HD of the animal, a process known as path integration [7]. However, constantly changing HD representations based on internal self-motion cues accumulate error [6,8,9], suggesting the need for a resetting process to help the system realign itself with the true HD of the animal. This resetting signal is likely to come from strong visual input indicating prominent landmarks by which the system may reorient [9].

The relative influence of visual and vestibular cues on HD cell responses is unclear. A number of studies have shown that, when these cues are in conflict, HD cell responses are primarily, although not completely, determined by vision. Cell preferred directions drift in the absence of visual cues and are reset by their return [6,10]. Despite visual dominance, it seems that cell preferred directions do not completely shift in response to movements of prominent visual cues, often undershooting the amount expected for total visual control [6,11,12]. However, other studies suggest that idiothetic (internally derived) information can override visual information, with cell preferred directions shifting in response to vestibular manipulations (even when this causes visual and vestibular information to be in conflict) [13], or failing to shift in response to shifts in visual landmarks [14].

An explanation for these mixed results is the effect of conflict size, that is, the distance between the directions indicated by idiothetic cues and by visual landmarks. This was explored in a study from Knierim *et al*. [8], who reported that idiothetic cues are a primary source of information for HD cell preferred directions, but that visual cues have an overriding corrective influence which depends on the degree of conflict between cue types.

Recent experimental work by Knight *et al.* [15] explored distance-dependent cue integration by systematically varying the discrepancy between visual cues and path integration mechanisms. Their data are explained fully in §1*a*. This paper is a theoretical counterpart to that of Knight *et al.* We present a hypothesized mechanism, at a neural network level, to explain HD system integration of conflicting information streams. Results gathered from a simulated instance of our hypothesis closely match experimental results, providing a strong indication of the plausibility of this account.

### (a) Experimental data of Knight *et al.* [15]

In the study of Knight *et al*., a rat freely explored a circular environment, which was in complete darkness barring a single orienting distal light cue. This provided a landmark to which the HD system could anchor. After 4 min of exploration, the light was extinguished and the rat explored in darkness for 10 s. A second light cue was then turned on for another 4 min, at *X*° conflict from the first light cue. This generated a conflict between the direction as indicated by the second light cue and the rat's internal sense of direction as indicated by path integration mechanisms.

Single-cell recording revealed that HD cells shifted their preferred firing direction in a manner dependent on conflict size, *X*°. Key features of these data to be modelled are: (i) For *X*° < 120°, cell firing directions were principally determined by vision. However, vision never totally dominated, with firing direction shifts undershooting the true value of *X*°. (ii) For *X*° > 120°, shifts were principally determined by path integration, again with an undershoot. (iii) Provided they have not previously been experienced as unstable relative to idiothetic information, visual cues can dominate preferred firing directions beyond *X*° = 120°, thought to be due to changes in saliency of visual information.

### (b) Model architecture

**Figure 1.**
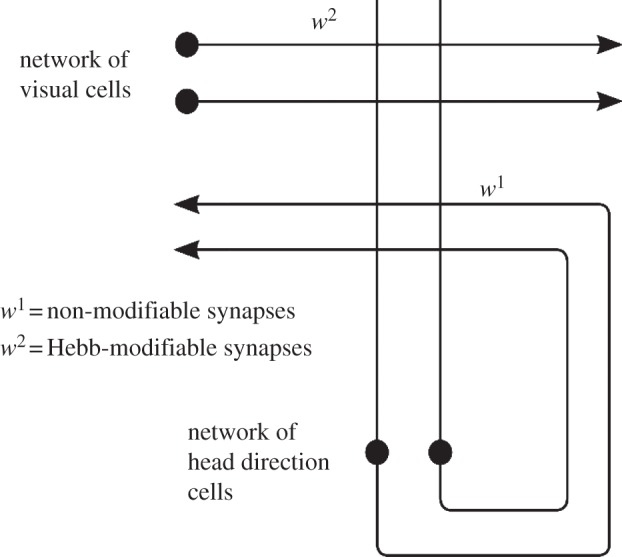
Network architecture, demonstrating connectivity both from the visual layer into the HD layer (*w*^2^ synapses) and within the HD layer itself (*w*^1^ synapses).

**Figure 2.**
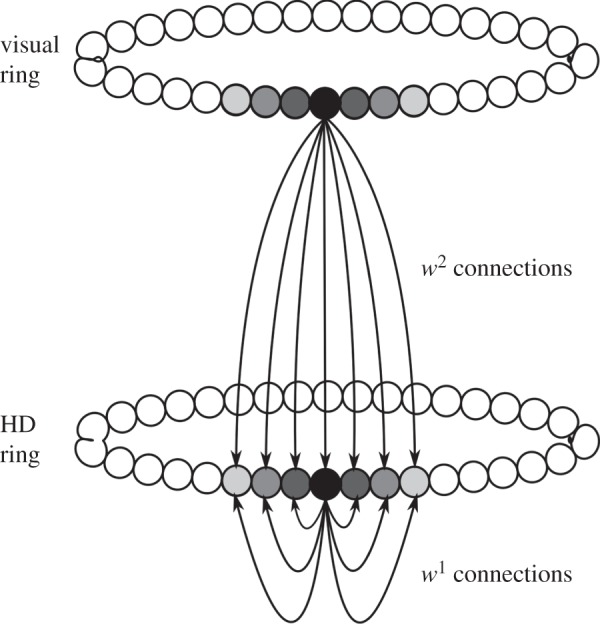
Network schematic, showing correspondence of activity packet locations in visual and HD rings.

Our model, similar to previous HD circuit models e.g. [16–18], consists of a ring of HD cells, simulated with a leaky-integrator firing rate model, in a continuous attractor neural network. HD cells receive input via plastic feed-forward connections projecting from a topographically corresponding region of a ring of visual cells. These visual cells encode the egocentric bearing from the agent to a visual cue in the environment. The visual cells are not explicitly modelled: instead, Gaussian activity packets are specified in the visual ring during simulation. Each HD cell thus receives non-modifiable recurrent collateral *w*^1^ connections and modifiable *w*^2^ feed-forward connections. Model architecture and schematic are given in figures 1 and 2. The model is simulated using a protocol resembling the experimental protocol of Knight *et al*. Full details of model equations and simulation protocols are given in the electronic supplementary material.

## 2. Experiment 1: cue conflict resolution

### (a) Resolving cue conflict: a hypothesis

Initially, each visual cell projects most strongly to a topographically corresponding HD cell. During a conflict between the bearing of the visual cue and the location of the packet of HD activity, a packet generated in the visual ring does not exactly correspond to that present in the HD ring. This visual activity packet will directly provide asymmetric stimulation to the existing HD cell packet. In the absence of learning, this would result in the initial HD packet moving to centre itself over the maximum of this visual input, which is the location of the visual landmark cue as specified on the visual ring [19,20]. However, experimental studies have shown that this does not happen. Instead, the memory packet within the HD ring appears to stabilize short of the location in the HD ring associated with the new location of the visual landmark. We have been able to explain these experimental observations by introducing learning into the connections from the visual cells to the HD ring.

**Figure 3.**
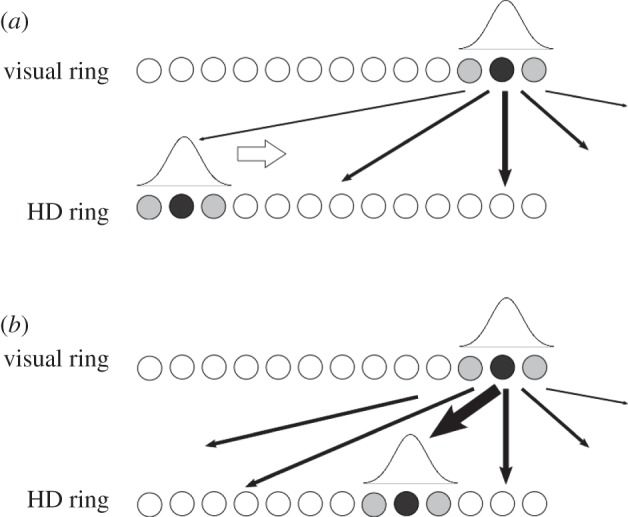
Diagram demonstrating hypothesis. Visual landmarks provide asymmetric input to the HD packet, shifting it through the network. Before learning (*a*), the strongest *w*^2^ weights (thickest lines) project to a corresponding location on the HD ring. After learning (*b*), weights are strengthened between the visual cue and every intermediate HD ring location, resulting in the strongest connections projecting to a new location on the HD ring.

The crucial aspect of this model is the plastic feed-forward connectivity between the visual and HD rings, which allows for variation in the final packet location within the HD cell ring. As the initial HD activity packet shifts to centre itself over the source of asymmetrical visual input, Hebbian learning is remapping the visual input onto the HD cell ring. Long-term depression (LTD), implicit within weight normalization [21], also occurs. As the activity packet moves around the HD cell ring, a strengthening occurs of the connections between the region of the visual ring that is active and the current location of the HD activity packet. Simultaneously, LTD weakens synapses between the currently active HD cells and non-firing presynaptic visual cells. This has the net effect of changing where in the HD ring the current visual activity maximally excites. The net effect of this remapping is to cause the packet to undershoot the location it would have reached were the original weight structure preserved, and to stabilize in a new position. Our hypothesized mechanism is represented graphically in figure 3.

### (b) Results

#### (i) Localized head direction ring remapping due to conflict

**Figure 4.**
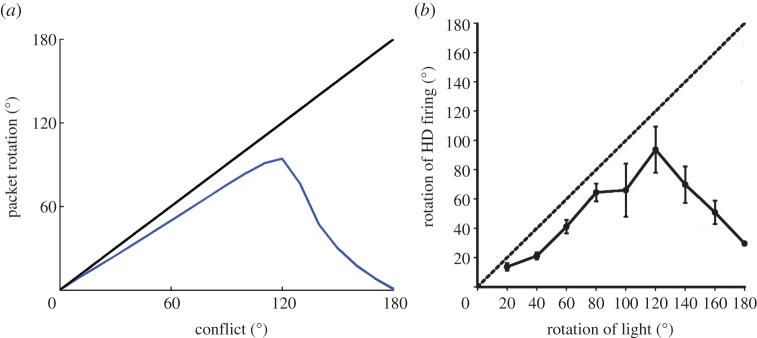
Summary of simulated results (*a*) showing final HD cell packet locations (blue line) for increasing conflict sizes, in contrast with expected packet location given total visual dominance without *w*^2^ synaptic learning (black line). For smaller cue conflicts, final packet location is determined by the visual cue, with an undershoot, until 120° conflict, after which final packet location is determined principally by the original location of the HD activity packet. This closely matches experimental results of Knight *et al.* ((*b*), with permission).

Figure 4 presents a summary of a block of 18 simulations using the model described above with model equations, parameters and simulation protocol given in the electronic supplementary material. As can be seen, there is a close match with previous experimentally derived results [15]. For conflicts below 120°, the final HD packet location is determined principally by the visual landmark cues. However, the combination of long-term potentiation and LTD within the feed-forward weights partially remaps the visual cue onto each intermediate location through which the packet moves. Due to the effect of this remapping, the packet motion is slowed down and undershoots the HD ring location corresponding to the visual cue. This undershoot increases with larger conflict sizes, as the packet is in motion over a larger distance, and thus there is a longer time interval over which more extensive remapping may occur. After a breakpoint at 120°, the conflict is large enough such that the influence of the visual landmark becomes weaker than the effect of the remapping process. This causes the final packet location to begin to be closer to the original HD cell layer representation than the new location indicated by the visual cue.

**Figure 5.**
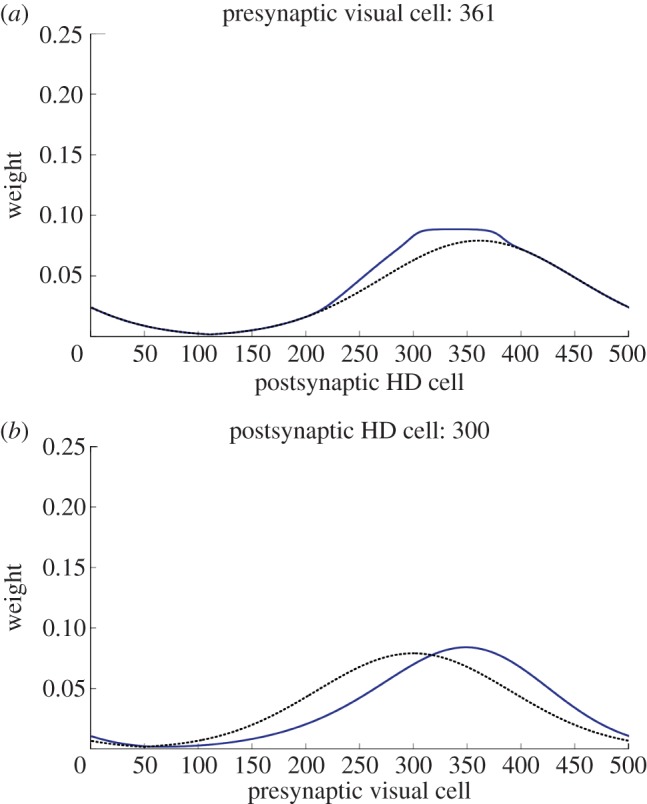
Feed-forward *w*^2^ weights from the perspective of the projecting visual cell at the centre of the conflicting visual landmark (*a*) and the receiving HD cell at the centre of the final HD packet (*b*), both before (black dashed) and after (blue) learning. Simulations cause a strengthening of weights from active visual cells to currently active HD cells. This causes the final HD location to receive maximal input from this new visual location (*b*).

Learning results in a change in structure from the original perfectly Gaussian *w*^2^ weight profile to one that reflects the remapping that has occurred during simulation. Figure 5 shows this change in the *w*^2^ weight structure during a typical simulation at 260° of conflict. The change occurs in weights that connect the active visual cells to HD cells representing locations between the initial and final packet locations, and thus is manifested as an asymmetrical distortion of the weight profile. As a result, the final HD cell packet location receives maximal input from the currently active visual cells.

**Figure 6.**
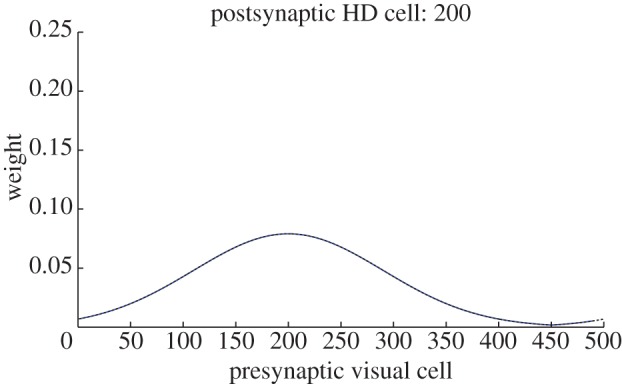
Feed-forward *w*^2^ synaptic weights from the perspective of a receiving HD cell outside cue conflict region, before (black dashed) and after learning (blue). Plots are coincident, demonstrating that simulation has not resulted in a change in *w*^2^ weight profile for this HD cell.

However, this change in weights only exists for HD cells within the regions through which the packet has moved. This is made clear by figure 6, which shows weights before and after the same simulation as figure 5 for postsynaptic HD cell 200, which is outside the region of the HD layer involved in the cue conflict. As can be seen, there has been no change in weights. This suggests that while some HD cells accurately change their preferred direction, this is only the case for part of the layer. This problem will be addressed further, and solved, in §3 of this paper.

**Figure 7.**
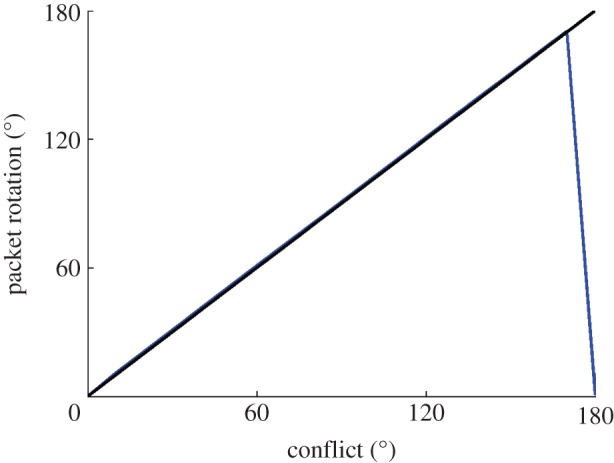
Results of simulations without learning in *w*^2^ synapses, conventions as in figure 4*a*. Without learning, no remapping occurs and the HD packet always moves to centre itself directly over input from visual cell layer, unless this input is symmetrical, as at 180° conflict.

The remapping shown in figures 4 and 5 is dependent on learning in the *w*^2^ synaptic connections. To confirm this, we ran additional simulations in which learning was switched off. These results with no learning are shown in figure 7. The visual landmark cue is not remapped onto any of the intermediate HD cell layer packet locations, because the feed-forward weight structure remains unchanged and, as expected, the packet simply moves to centre itself directly over the input provided by the visual landmark cue. At 180° conflict, just as with simulations run with plastic feed-forward weights, the visual cue is located such that it provides symmetrical input to the initial location of the HD cell packet, which therefore does not move.

#### (ii) Salience of visual cues

**Figure 8.**
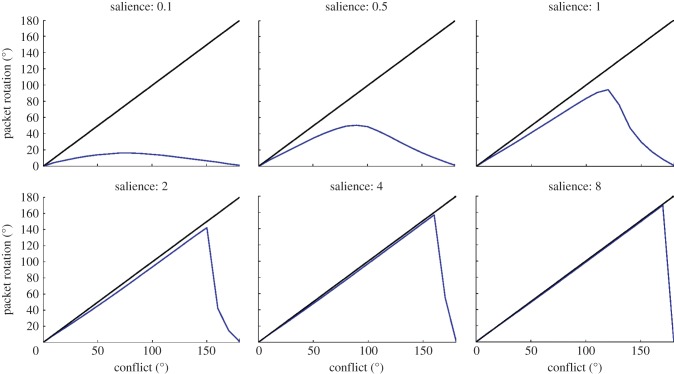
Results for simulations with varying values of salience, **ϕ**_2_. At lower salience, the system favours idiothetic information, with larger undershoots and earlier breakpoints. As salience increases, visual cues dominate cue conflict, with larger breakpoints and smaller undershoots, until final packet location is determined almost entirely by visual input for **ϕ**_2_ = 8 and above.

Results from the Jeffery laboratory (see §1*a*) suggest that, in addition to conflict size, saliency of visual cues may have an effect. Experimentally, this seems to be the case where visual landmark cues become less salient when they have been learned as unstable, that is, lack correspondence with idiothetic information (see fig. 5 [15]). In cases where visual landmarks are less salient, it seems that shifts in cell preferred directions are governed increasingly by idiothetic information. This result has been replicated in the model. Presented in figure 8 are summary results for a series of simulations with identical parameters and varying conflict size. However, for each simulation we vary the overall scaling of feed-forward visual weights onto the HD layer to represent changes in salience of visual information.

For low values of salience, the visual landmark has poor control over the final packet location, and thus conflicts tend to be resolved closer to the initial HD layer memory packet. As the strength of the visual cue is increased, the relative effect of remapping is weakened and final packet locations are determined increasingly by visual cues. This is reflected in a breakpoint which is progressively later, and initial undershoots which are progressively smaller, with increasing values of the scaling parameter.

## 3. Experiment 2: isomapping across the entire head direction ring

The previous model provides a clear explanation of a biologically plausible neural mechanism that could underpin cue conflict resolution behaviour. However, this explanation is limited in that only a subregion of cells within the HD layer updates their preferred firing directions, resulting from a change in the visual location to which cells respond maximally. Real HD cells in the rat tend to shift preferred firing directions in register, with cells shifting by similar amounts in response to environmental manipulations [6,22]. Thus, the angular distance between preferred firing directions of individual HD cells is preserved across changes of environment: a property known as *isomapping*. In this section, we present results from an extended simulation paradigm making use of path integration mechanisms to allow all HD cells within a layer to ‘inherit’ the shift in preferred firing directions of the initial subset of remapped cells.

### (a) Isomapping: a hypothesis

After the initial cue conflict, simulated in §2, weights have been updated from cells representing the location of the visual landmark to cells in the subregion of the HD layer through which activity has propagated, as shown in figure 3. This results in these HD cells responding maximally to different egocentric bearings to the visual cue and resulting in a shift in preferred firing directions. However, these changes have only occurred for a subset of HD cells within the ring, and most cells retain their original configuration with visual space.

**Figure 9.**
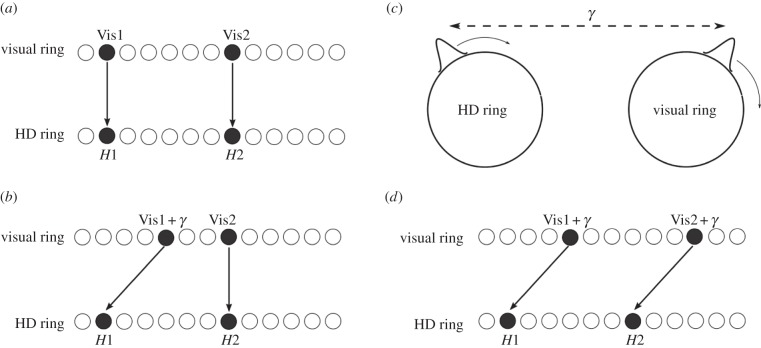
Isomapping theory. (*a*) Both HD cells receive from corresponding visual ring locations. (*b*) After initial cue conflict, cells within cue conflict region (*H*1) change preferred visual ring location, but other cells (*H*2) do not. (*c*) During subsequent agent rotation and path integration, the new relationship between visual and HD packets is learned around the whole HD ring, as HD and visual activity packets shift in lockstep. (*d*) After the agent rotates several times, all cells across the HD layer shift their preferred firing directions by similar amounts.

Consider just two HD cells, *H*1 and *H*2. *H*1 is the centre of the final HD packet location after cue conflict, while *H*2 is an arbitrary cell that has not been active at any point during cue conflict. Before simulation, *H*1 and *H*2 receive maximally from visual locations Vis1 and Vis2, respectively (figure 9*a*). After cue conflict, feed-forward weights have been updated such that *H*1 now receives from location Vis1 + *γ*, where *γ* reflects the disparity between the HD packet location and the visual packet location (i.e. the amount of undershoot). Thus, *H*1 has shifted preferred direction by *γ*. Conversely, *H*2 has never been active, and thus no update has been made to the weights projecting to this cell (figure 9*b*).

If the agent then rotates on the spot, the activity will continue to propagate through the visual ring. Assuming then, that path integration system input to the HD cells is capable of driving activity through the HD cell layer at the same speed as the agent's rotation, the HD activity packet will move at the same speed as the visual packet (figure 9*c*). This will preserve the disparity of *γ* between HD and visual locations. When cell *H*2 becomes active, it will be coactive with visual location Vis2 + *γ*, and thus Hebbian learning will occur to strengthen projections from Vis2 + *γ* to *H*2. This means that *H*2 will have shifted preferred direction by *γ*, identically to *H*1. As the agent rotates on the spot, continued Hebbian weight updates performed on *w*^2^ connections will result in HD cells across the entire layer learning to receive maximal input from a visual location displaced by *γ* from their original HD preferred direction, exactly as for the initial subset of HD cells whose preferred directions shifted after initial cue conflict. This results in all HD cells inheriting a change in preferred directions, and thus leads to isomapping (figure 9*d*).

### (b) Results

#### (i) Isomapping around the entire head direction ring

**Figure 10.**
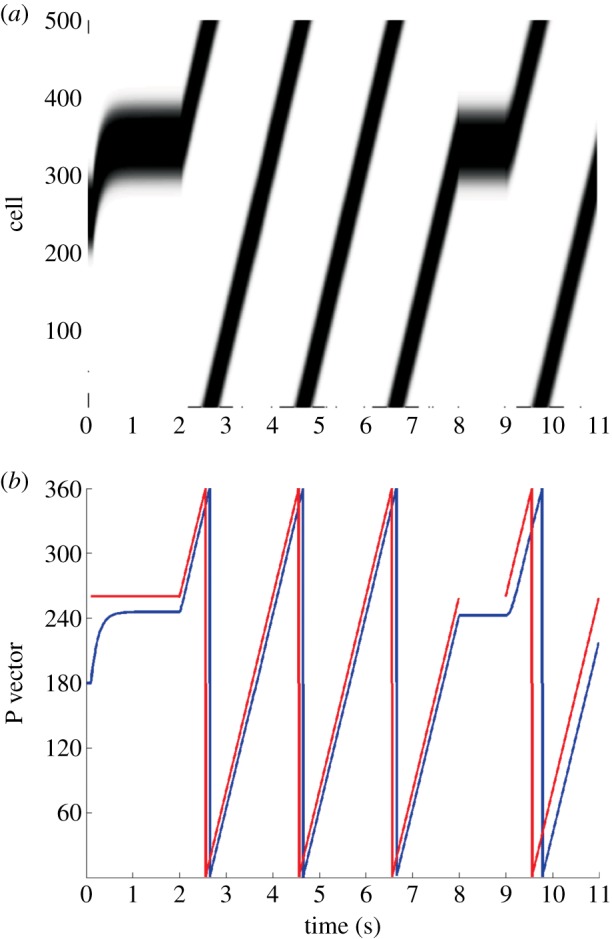
Full isomapping simulations begin with 2 s of cue conflict for stationary agent. This is followed by agent rotation for a further 6 s, during which the entire HD cell layer remaps onto the visual ring. Agent is then stationary for 1 s, before a 2 s period during which visual activity is propagated through the ring to test the consistency of the new visual-HD weight profile. Summary results show HD layer firing (*a*) and relative locations (P vectors) of HD (blue) and visual (red) packets (*b*) during simulation.

Figure 10 presents summary graphs of a simulation run for a conflict size of 80° between the visual cue and initial HD packet location. In figure 10*a*, we see the firing rates of the entire HD cell population over time. Figure 10*b* shows the locations of the HD (blue) and visual (red) packets over time.

During the first two seconds of cue conflict, we see that the conflict is resolved in a similar manner to experiment 1: a final HD cell packet location which undershoots that of the visual packet by a similar amount (14.13°) to results given in previous simulations (14.05°). This is reflected in figure 10*b*, with the HD packet location pulling towards, yet stopping short of, the visual packet location. This gives a final HD packet location shifted 65.87°.

From seconds 2 to 8, we see the packets move through the visual and HD rings at angular velocity *V*. During this period, we see in figure 10*b* the fact that both the visual and HD packets appear to be moving, and that the disparity between them introduced by cue conflict is preserved through all locations. Following this rotation period, visual input is removed and the HD cell activity packet is maintained in position through recurrent collateral synapses.

**Figure 11.**
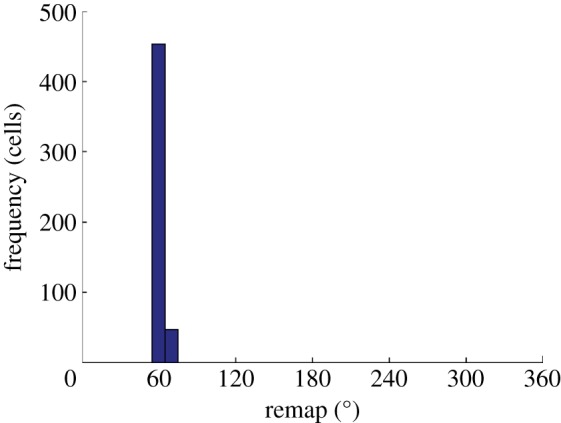
Histogram, with bin size of 7.2°, showing change in preferred direction of all HD cells after cue conflict and path integration. The narrow distribution demonstrates isomapping around the entire ring of HD cells after agent rotation and path integration have repeatedly driven activity through the entire HD layer.

From seconds 9 to 11, during a period where no further learning takes place and HD activity is influenced solely by visual input, we see that the HD cell activity accurately tracks visual input, reflected in a consistently sized separation of HD and visual packets in figure 10*b*. Together, these observations indicate that the remapping of feed-forward weights has been coherent. This is supported by figure 11, which shows a histogram plotting the amount of remap displayed by cells within the HD layer, with a bin size of 7.2°. The remap is calculated as the difference between the initial and final directions of receiving weights for a given cell. In this case, with a conflict size of 80° and a final HD packet location that undershoots the visual location by 14.13°, the expected remap size would be equal to the amount by which the HD cell packet has shifted, 65.87°. All cells are tightly clustered close to the expected remapping value of 65.87°, with a population mean of 61.94° and an s.d. of 1.84°. This effect was found not to be direction dependent and an identical cue conflict performed in the opposite direction resulted, after cue conflict, in an activity packet undershooting by approximately 15° and, after path integration, in a coherent remapping reflecting this undershoot (mean = 72.21°, s.d. = 1.82°).

**Figure 12.**
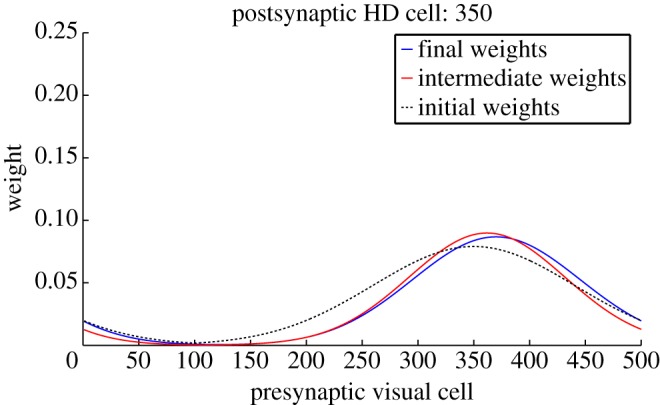
Effect of learning during isomapping on *w*^2^ weights onto an HD cell inside the cue conflict region. Weights are shown before learning (black dashed), after cue conflict (red) and after isomapping (blue). This demonstrates intermediate remapping during cue conflict, which is consolidated during path integration.

**Figure 13.**
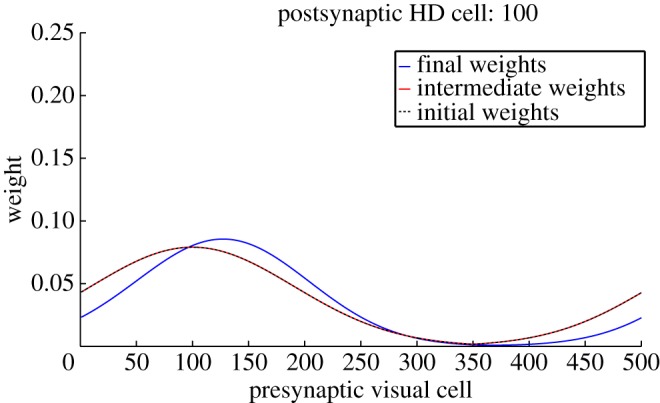
Effect of learning during isomapping on the *w*^2^ synaptic weights onto an HD cell outside the initial cue conflict region. Conventions are the same as figure 12. Although intermediate remapping is absent, shown as coincident initial and post-cue conflict weight profiles, path integration allows this cell to inherit the remapping of cells within the cue conflict region.

Figures 12 and 13 show feed-forward weights before simulation, after cue conflict, and after path integration for an HD cell within the cue conflict region, and a cell outside the conflict region. Crucially, both cells show a difference between initial (black dotted) and final (blue) weights. Of particular interest are the intermediate weight values (red). We can see that, for a cell in the cue conflict region, intermediate weights are partially remapped. However, this remapping is inherited by the rest of the HD layer only after rotation and path integration. This supports our hypothesis of a two-stage remapping process.

#### (ii) Salience of visual cues

**Figure 14.**
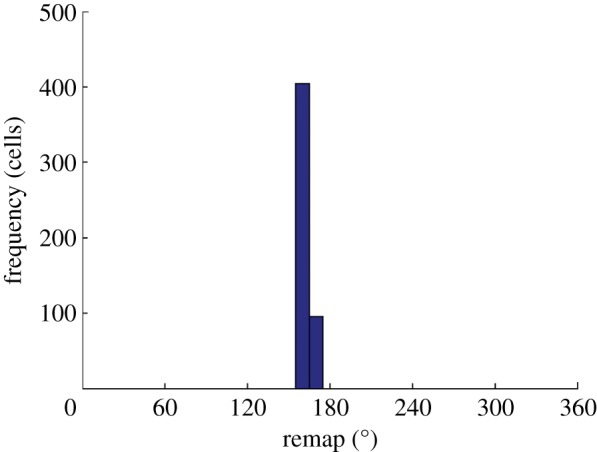
Histogram of changes in preferred direction for all HD cells, as in figure 11, for a maximally salient visual landmark at a large separation of 170°. Total cue capture is demonstrated.

Similar to experiment 1, we tested isomapping performance with a maximally salient visual landmark cue at a large conflict size of 170°. Ordinarily, conflicts this size would result in a final HD packet location which is dominated by idiothetic information. However, maximally salient visual cues should result in a remap of 170° to match the conflict size. Figure 14 presents, similar to figure 11, a histogram showing the amount of remap for the HD cell layer between the beginning and end of simulation. A coherent remapping has taken place, with most cells clustered around a mean value of 161.2° with an s.d. of 4.21°. This demonstrates how a salient visual cue may dominate cue conflict, and in turn cause HD cells to completely remap on the basis of such a cue.

## 4. Discussion

The model presented in this paper represents a computational account of how internal self-motion and external visual sources of information interact in the HD cell system during cue conflicts, resulting in changes of HD cell preferred directions with respect to a visual cue. These changes constitute a compromise between these two information sources.

We propose that these changes in preferred HD happen as part of a two-stage process. Initially, only a portion of cells in the HD layer change their preferred directions in response to cue conflict. However, when the agent begins to move after the intitial cue conflict resolution, the egocentric bearing to the visual landmark changes and idiothetic input acts to shift the HD activity packet in a manner consistent with the changes in landmark bearing. This updates HD preferred directions for all cells, reliant on accurate path integration mechanisms. This two-stage process represents a testable hypothesis. Due to isomapping, shifts in the firing properties of the entire HD cell layer are typically inferred experimentally from the shifts of an individual cell or small group of cells [6,22]. In future, recordings of ensembles of HD cells may well prove informative as to the validity of our hypothesized two-stage mechanism. We note that there has been little study of HD cells in terms of entire populations thus far [23,24].

Certainly, the idea of the HD system reorienting over a short period of time in response to environmental manipulations is supported by experimental evidence regarding the time scale over which cue conflicts involving a learned visual cue are resolved [25]. Although the results presented in this paper represent conflict resolution within a minute, it is more than plausible that such a mechanism could work over a reduced time course in a spiking, rather than rate-coded, model [26]. We should note, for clarity, that we address specifically the speed at which HD cell activity can be influenced by an established visual landmark, rather than time taken to establish a stable visual landmark as a reliable cue. The latter, in terms of our model, would represent learning of the *w*^2^ connectivity, which is instead explicitly initialized in our simulations.

The second finding of this study is the effect of cue salience. It appears that more salient visual cues exhibit stronger cue control, with cue conflict therefore being resolved more in favour of visual landmarks and HD cells subsequently shifting their preferred directions further in favour of the visual cue. This is demonstrated in the second experiment of Knight *et al.* [15], in which the amount of compromise shown by HD cells was experience dependent. The idea that only visual cues with a stable relationship to idiothetic information can form landmarks is evidenced in the literature to date [9,27]. For example, Knierim *et al*. [28] demonstrated that periods of disorientation stopped the establishment of stimulus control by visual landmarks. Equivalent findings are available for place cells [29].

These findings are entirely consistent with the idea of salience presented in this paper: less salient visual cues are weakened as inputs to the model, resulting in weaker cue control. Salience affects how well a visual cue is learned as a stable landmark. In terms of the model, this would affect the strength and topopgraphical organization of the *w*^2^ weight profile. While we simulate the effect of salience via a uniform downweighting of prespecified *w*^2^ connectivity, visual cue stability likely has an effect on how initial *w*^2^ connectivity is formed. These changes in salience represent *w*^2^ plasticity over a longer timescale. By contrast, visual to HD remapping and isomapping are relatively more labile, occurring within a trial rather than across trials. This shorter term plasticity would happen on top of an initialized *w*^2^ profile. A future modelling direction would be to explicitly simulate the development of the *w*^2^ weight profile in a novel environment. This would, in essence, encapsulate the concept of salience without, as in the current model, the need for an explicit parameter: only stable visual cues would form ordered *w*^2^ connectivity, and thus act as landmarks.

Interestingly, landmark stability appears to be a mechanism by which the HD cell system is able to differentiate between distal and proximal visual landmark cues [30–32]. This distal–proximal distinction has been successfully modelled, and demonstrated to depend on the fact that distal cues maintain a stable relationship with idiothetic information as the agent explores an environment but proximal cues do not [33]. These findings could be explained by our conception of salience. However, we note that such changes in salience are not limited to landmark stability, and indeed may be modified on behavioural or motivational terms, e.g. a visual landmark which has previously been associated with reward might demonstrate cue control more readily than a landmark of neutral valence.

Our model makes use of an abstracted term representing idiothetic input to HD cells, used to shift activity through the HD layer at the correct speed during simulated path integration. This provided a simple and uncluttered explanation of our hypothesized remapping mechanism in action during cue conflict. Future simulations, however, could well incorporate detailed neuronal structures for performing path integration [34,35].

The simulation paradigm used involves only rotation in a single direction, and distinctly separated cue conflict and path integration phases (by virtue of the simplifying assumption we make that the agent remains stationary during cue conflict). Separating cue conflict and path integration when simulating changes in cell preferred direction is necessary to elucidate the mechanism by which cell preferred directions are updated. However, *in vivo*, these two stages would overlap. One can see how the mechanism described above could be adapted to more ecologically valid simulation paradigms, with remapping due to cue conflict being inherited across an entire HD cell layer even as this cue conflict is being resolved, and with the final shift in preferred directions remaining a compromise between visual and idiothetic information sources.

Although this work successfully models experimental data, we note that what has been replicated is an average of the data recorded across all rats. Individual rats show a degree of variation in cell responses, in terms of both shift amount and latency. However, exploration of parameter values (not reported here) demonstrates that this model is capable of a broad range of behaviours. For instance, as demonstrated by varying salience (figure 8), strong inputs can cause complete light capture of HD cell preferred directions (as seen in rat 321 by Knight *et al*. [15]). Thus, biological correlates of these parameters, which could vary between rats, can account for this heterogeneity.

To summarize, we present here simulation results aimed at demonstrating a theoretical account of cue averaging in the rodent HD cell system. We hypothesized that cue averaging is critically dependent on plastic remapping of visual information onto the HD cell system. For a subset of HD cells, this plasticity results in a shift in preferred firing directions and prevents visual information from dominating cue conflicts, with shifts in cell preferred directions being dependent on the size of disparity between idiothetic and visual information sources. We also suggested that the initial remapping for a subset of cells during cue conflict may be inherited by other cells in the layer during subsequent movement and path integration. Finally, we demonstrated how the influence of the visual landmark could be weakened by reducing its salience. This may well help to explain such phenomena as weakening of landmark stimulus control after disorientation, and the separation of distal and proximal landmarks based on visual motion cues. More generally, our results provide a mechanism for reliability-weighted cue averaging that may pertain to other neural systems in addition to the HD system.
